# Human Umbilical Cord–Derived Cells Therapy for Hypoxic-Ischemic Encephalopathy: A Systematic Review and Meta-Analysis of Animal Models

**DOI:** 10.1155/ijpe/1111052

**Published:** 2025-09-15

**Authors:** Yincong Chen, Yanling Lu, Yueqin Ding, Jianping Chen, Kuihuan Tan, Jian Ding, Linqi Huang, Peisi Chen, Zhifeng Chen

**Affiliations:** Department of Pediatrics, The Tenth Affiliated Hospital, Southern Medical University (Dongguan People's Hospital), Dongguan, China

**Keywords:** cognitive, hypoxic-ischemic encephalopathy, infant, neurological function, sensorimotor, umbilical cord

## Abstract

**Introduction:** Neonatal hypoxic-ischemic encephalopathy (HIE) is a severe neurological disorder associated with high mortality and long-lasting complications in survivors. Umbilical cord–derived cells have emerged as promising therapeutic candidates, demonstrating positive results in experimental HIE research. This review systematically evaluates the current preclinical evidence on the efficacy of umbilical cord–derived cells in animal models of HIE, focusing on their effects on neurological function and identifying research gaps and constraints that need to be addressed for future preclinical and clinical studies.

**Methods:** The present systematic review and meta-analysis, conducted in accordance with the Systematic Review Protocol for Animal Intervention Studies, synthesized the available evidence on the efficacy of umbilical cord–derived cells. Relevant studies were searched for in Medline's PubMed, Web of Science, Embase, and Cochrane databases up to May 1, 2024. Papers that included the interventional use of UC-derived cells were considered, regardless of the source, dose, timing, and frequency. Nevertheless, studies involving modified UC-derived cells or combination therapies were excluded. Functional neurological outcomes were extracted for meta-analysis to calculate standardized mean difference (SMD) with 95% confidence interval (CI), using a random effects model. The risk of bias was evaluated using SYRCLE. Forest plots and funnel plots were utilized to evaluate potential publication bias.

**Results:** Twelve studies were incorporated into the systematic review. The meta-analysis indicated a significant positive impact of umbilical cord–derived cells on neurobehavioral outcomes post-HIE injury. Sensorimotor function showed an improvement of 0.80 SMD (95% CI, 0.58–1.03) in the negative geotaxis test, while cognitive function demonstrated a 1.44 SMD (95% CI, 1.21–1.67) improvement in the water maze test. The subgroup analysis demonstrated heterogeneous effect sizes contingent on distinct study characteristics, including animal age, cell type, cell dosage, delivery method, and timing method.

**Conclusions:** Overall, these findings suggest a promising role for umbilical cord–derived cells in preclinical HIE studies. The treatment with umbilical cord–derived cells exhibited enhanced functional outcomes, showing promise for future translational research. Despite limitations such as bias risk and heterogeneity affecting the meta-analysis robustness, our results align with existing literature in this research domain.

**Trial Registration:** ClinicalTrials.gov identifier: CRD42024551469

## 1. Introduction

Neonatal hypoxic-ischemic encephalopathy is a clinical syndrome stemming from perinatal factors, including intrauterine distress and neonatal asphyxia, which has high morbidity and mortality rates [1]. Unlike similar injuries in the mature brain, it shows more severe inflammation and apoptosis. In clinical manifestation, the majority of affected newborns are likely to suffer from persistent neurological impairments, including auditory and visual deficits, developmental delays, cerebral palsy, and seizures [2–4]. Currently, therapeutic hypothermia is the only recognized effective treatment for low severity of HIE in full-term infants [5]. Nevertheless, hypothermia has not been demonstrated to improve outcomes associated with severe HIE [6]. Moreover, therapeutic hypothermia, regardless of duration, is linked to various complications such as coagulation disorders, exacerbated acidosis, electrolyte disturbances, pancytopenia, pulmonary hypertension, and arrhythmias. Consequently, there is an urgent requirement for novel treatment approaches aimed at addressing HIE.

In the past decade, human umbilical cord–derived cells (UC-derived cells) have emerged as a promising therapeutic approach for managing HIE. Human UC-derived cells consist of a large number of monocytes (MNCs) (40%), lymphocytes (40%), neutrophils (10%), and a remaining 10% composed of mesenchymal stem cells (MSCs), endothelial progenitor cells, and pluripotent stem cells [7]. Previous studies have confirmed that MNCs in UC-derived cells contain approximately 3%–10% immature progenitor cells [7, 8]. Another study found that the transplantation of UC-derived MSCs or cord blood–derived MNCs following hypoxic-ischemic injury can prevent neuronal loss and significantly improve functional brain outcomes [9]. Furthermore, these cells can be easily collected and stored for future therapeutic and research purposes. Additionally, the risk of immune rejection following the transplantation of UC-derived cells is lower compared to bone marrow–derived MSCs, as the levels of HLA antigens remain relatively low [10].

Preclinical studies utilizing animal models of HIE have demonstrated that UC-derived cells enhance functional recovery in treated groups. The therapeutic potential of UC-derived cells in neurological disorders stems from their ability to restore cellular energy, attenuate the inflammatory response, inhibit apoptosis, and secrete various neurotrophic factors and growth factors [11]. The recent validation of using autologous cord blood for treating HIE in human infants establishes a foundation for forthcoming randomized controlled trials [12]. Notwithstanding the advancements, there has been no attempt to consolidate the existing literature on UC-derived cell therapy for HIE treatment. This paper systematically evaluates the effectiveness of UC-derived cells as a therapeutic intervention in preclinical HIE models, with the aim of informing the design of future preclinical investigations and clinical trials in this research domain.

## 2. Methods

Our methods follow the Preferred Reporting Items for Systematic Reviews and Meta-Analyses (PRISMA) guidelines, a rigorous and standardized approach for evaluating preclinical animal intervention studies [13]. Our protocol has been registered on PROSPERO on August 21, 2024.

### 2.1. Literature Search

In summary, we conducted a literature search up to May 1, 2024, using Medline's PubMed database, Web of Science, Embase, and Cochrane. The search terms comprised “Human umbilical cord cells,” “neonatology,” “hypoxic ischemic encephalopathy,” “preclinical,” and their synonyms. The search formula is (“Hypoxia-Ischemia, Brain”[Mesh]) AND(“Bloods, Umbilical Cord”[Mesh]) AND (“Fetal Blood”[Mesh]OR“ Wharton Jelly”[Mesh])AND(“Nervous System Physiological Phenomena”[Mesh]). Before the screening process, duplicate studies were removed manually from the search results. Two investigators (Yanling Lu and Kuihuan Tan) independently screened titles and abstracts, followed by a review of full texts. In cases of disagreement, a third investigator (Yueqin Ding) was consulted for resolution. Furthermore, we conducted manual searches of reference lists from included studies and relevant reviews to identify additional studies for inclusion.

### 2.2. Inclusion and Exclusion Criteria

In this study, HIE is characterized as a sudden cessation of cerebral blood flow, oxygen, and nutrient supply. Experimental models commonly simulate HIE by ligating or occluding the carotid artery. Studies meeting the inclusion criteria assessed the impact of UC-derived cell intervention on functional neurological outcomes in validated preclinical, in vivo neonatal HIE models. In the context of rodents, neonatal models encompass subjects under 10 days old [14]. For the purpose of this review, MSCs will be defined according to the guidelines set by the International Society for Cellular Therapy (ISCT) [15]. Papers including interventional use of UC-derived cells were considered regardless of source, dose, timing, and frequency. However, studies involving modified UC-derived cells or combination therapies were excluded. Labeled UC-derived cells (e.g., iron oxide particles and green fluorescent protein) were utilized for tracking and locating cell distribution. The treatment group received UC-derived cells after HIE induction, whereas the control group experienced HIE injury but received a solvent or placebo (e.g., phosphate-buffered saline and normal saline).

### 2.3. Primary and Secondary Endpoints

Our main focus was on assessing functional neurological outcome, which is evaluated through cognitive or sensorimotor testing after the induction of HIE. The primary endpoint in this study was defined as the functional neurologic outcome, assessed through cognitive or sensorimotor testing postinduction of HIE. Cognitive tests (e.g., water maze) and sensorimotor tests (e.g., negative geotaxis) commonly used in preclinical studies of neurologic damage were included. Studies lacking data on the primary outcome during the data extraction phase were excluded. Although not mandatory for inclusion, lesion size, a secondary outcome, was reported in many selected studies. The primary focus of this review is on the analysis of the main endpoint, while the results from the analysis of secondary endpoints will be included in a future publication.

### 2.4. Data Extraction

Data were independently collected by two investigators (Yanling Lu and Jianping Chen) and cross-checked for accuracy. In cases of discrepancies, a third investigator (Jian Ding) was consulted for resolution. The extracted data encompassed details on the study design (such as sample size), animal characteristics (e.g., age), intervention specifics (including source, dosage, method of delivery, timing, and frequency), and outcome measures relevant to the main endpoint. Original data, such as mean values with standard errors, were extracted from graphs and plots using GetData graph digitizer Version 2.26 in cases where precise values were not explicitly provided in the source article. In situations where studies presented multiple intervention variations (e.g., different doses, days, or frequencies), each set of results was treated separately.

### 2.5. Risk of Bias

Two researchers (Yanling Lu and Peisi Chen) independently assessed the potential for bias in each study using SYRCLE's risk of bias tool, and a third researcher (Linqi Huang) was consulted to resolve any disagreements [16]. The tool includes 10 evaluation areas related to selection, performance, detection, attrition, and reporting biases. Each area was categorized as having low, high, or unclear risk of bias based on specific questions within the tool. A “yes” response denoted a low risk of bias, while a “no” response indicated a high risk of bias. Studies lacking explicit method descriptions were classified as “unclear.”

### 2.6. Data Analysis

A random effects meta-analysis was conducted to generate forest plots. The standardized mean difference (SMD) and corresponding 95% CI were utilized to estimate the effect size of UC-derived cells on functional neurological outcomes following HIE. SMD, an appropriate measure for continuous data, was computed by dividing the mean difference in each study by the respective standard deviation. In the case of papers with multiple relevant and distinct treatment arms, each treatment arm was recorded as an individual entry in that forest plot. Separate analyses were performed to determine the effect sizes for sensorimotor function and cognitive function. Additionally, a combined analysis of all neurobehavioral studies was conducted to assess the overall effect size.

Statistical heterogeneity was assessed using the *I*^2^ statistic, where *I*^2^ > 50% indicates substantial heterogeneity. Subgroup analysis was conducted to explore significant sources of heterogeneity. Publication bias was assessed by visually inspecting funnel plots for asymmetry. The analysis was carried out using Review Manager (RevMan) (Windows 11), Version 5.4, developed by The Cochrane Collaboration, 2020. All statistical tests were two-tailed, with significance defined as *p* < 0.05.

## 3. Results

### 3.1. Study Selection

**Figure 1:**
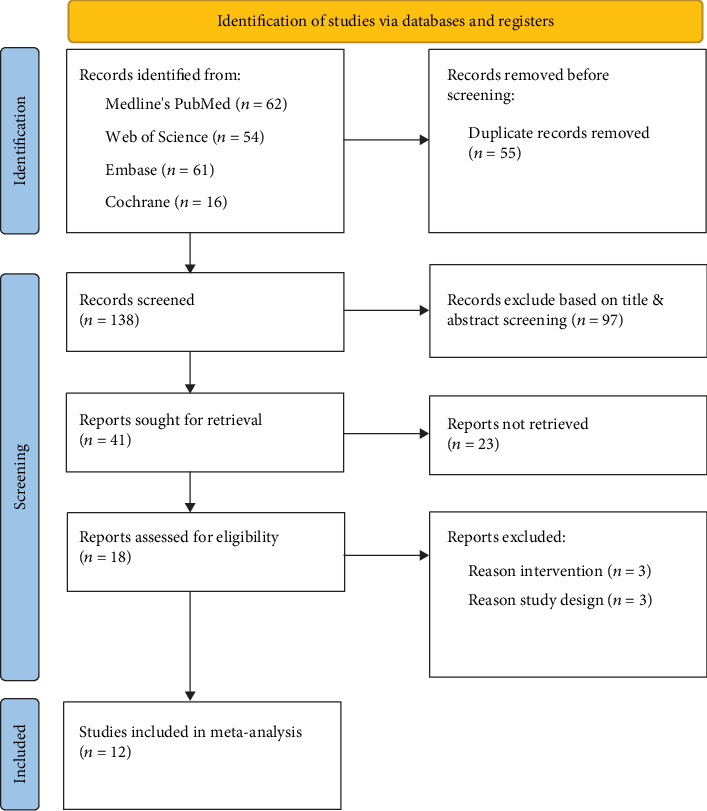
PRISMA diagram detailing study selection process.

The literature search yielded 193 results using the specified search terms. Following manual removal of duplicates, 138 studies were retained. Following screening of titles and abstracts, 85 studies centered on the therapeutic use of UC-derived cells in HIE were identified for full-text evaluation. Ultimately, 12 publications meeting the predefined eligibility criteria and reporting functional neurologic outcome as the primary endpoint were included (Figure 1).

### 3.2. Study Characteristics

The characteristics of the included studies are presented in Table 1. All the research encompassed in this review was released from 2013 to 2024. The timing of brain injury varied from postnatal Day 7 (PND7) to PND10, corresponding to the developmental stage of a human neonate. Intraperitoneal injection emerged as the predominant delivery route, with dosages varying between 200 thousand cells and 20 million cells. In terms of intervention timing, the majority of studies (80%, *n* = 516) administered cells within 24 h of HIE, followed by 14% (*n* = 90) within 48 h and only 1% (*n* = 9) within 72 h. A small percentage of studies (5%, *n* = 3) used multiple doses. All included studies assessed functional neurologic outcomes. The most frequently utilized approach for evaluating sensorimotor function was the negative geotaxis test, whereas the assessment of cognitive function predominantly involved the use of the water maze. Only one study mentioned composite scores such as the Longa score [26].

### 3.3. Risk of Bias

**Figure 2:**
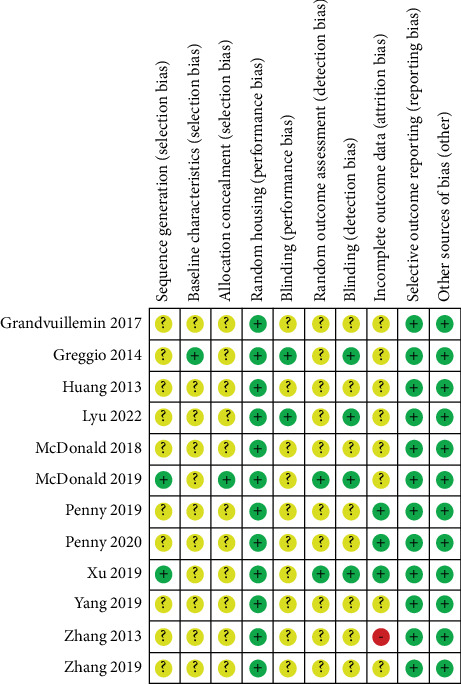
Systematic Review Center for Laboratory Animal Experimentation (SYRCLE) risk of bias assessment.

The SYRCLE risk of bias tool was used to evaluate bias risk in all 12 studies that were included in this review (Figure 2) [16]. While the included studies were all randomized controlled trials, only one study reported randomizing animals using a random number table, resulting in an unclear risk of selection bias across a large proportion of the domains. Similarly, only one study each reported baseline characteristics and allocation concealment, further contributing to the unclear risk of selection bias. All studies indicated that animals were housed in a random manner; however, only 17% (*n* = 2) of the studies disclosed blinding of caregivers and investigators to the intervention received by each animal. In terms of outcome assessment, 17% (*n* = 2) of the studies indicated that the outcome assessment was conducted randomly, and 33% (*n* = 4) mentioned that the outcome assessor was blinded. One study reported a small amount of missing data, while three studies had no missing data; the remaining studies did not mention any missing data. No further biases, such as financial support from the industry, conflicting interests, or not publishing in a peer-reviewed journal, were found apart from those addressed by the SYRCLE risk of bias tool.

### 3.4. Stratified Meta-Analysis: Functional Neurologic Outcome

**Figure 3:**
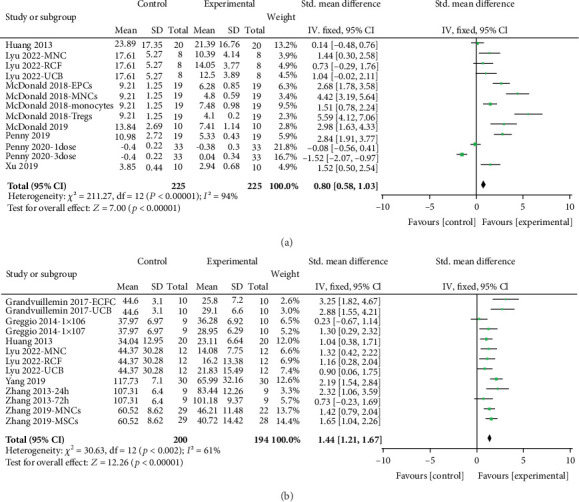
Effect size of UC-derived cells cells across functional neurologic assessments from included studies. Forest plots demonstrating SMD and 95% CI for (a) negative geotaxis test and (b) water maze test.

**Figure 4:**
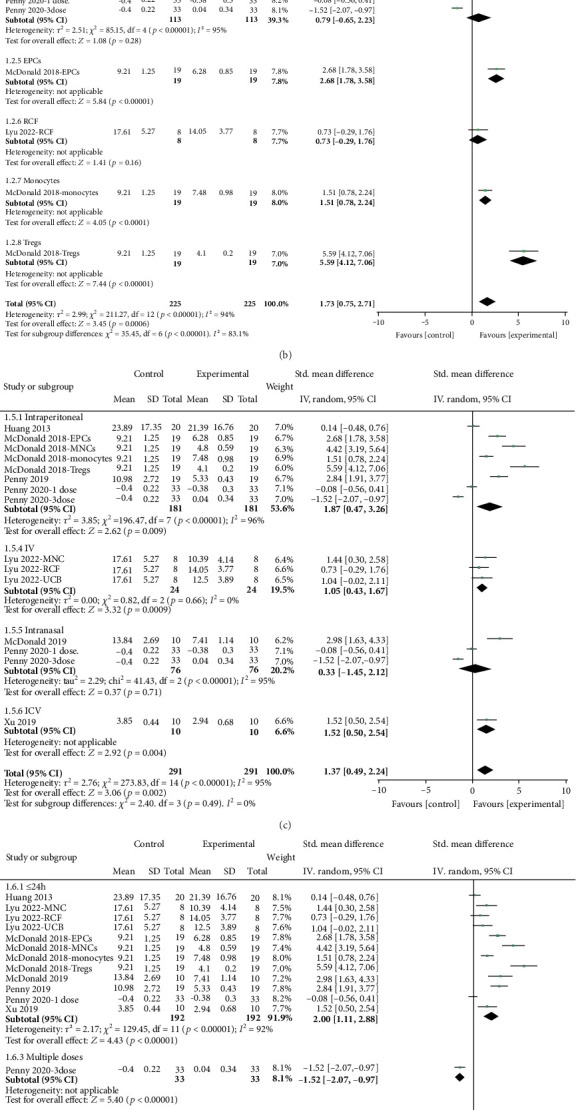
Stratification of estimated effect size for sensorimotor function. Forest plots demonstrating SMD and 95% CI for (a) age injury induced, (b) cell type, (c) route of administration, (d) cell administration time postinjury, and (e) total cells per dose.

Sensorimotor: Sensorimotor outcomes were evaluated using the negative geotaxis test. The overall performance on this test showed a substantial improvement, with a SMD of 0.80 (95% CI, 0.58–1.03; 7 studies and 13 entries; Figure 3a). However, significant heterogeneity was observed between the study groups (*I*^2^ = 94.0%; *p* < 0.00001). Nine out of the 13 interventions demonstrated effect sizes exceeding 0.8 SMD, with the most substantial effect size recorded at 5.59. Subgroup analysis considering animal model, study design, and intervention characteristics revealed significant variability in effect sizes, as presented in Figure 4. Notably, interventions conducted on PND7 exhibited enhanced sensorimotor performance (2.20; 95% CI, 1.17–3.23). The most substantial effect size was observed when the administered cells were Tregs (5.59; 95% CI, 4.12–7.06), delivered intraperitoneally (1.87; 95% CI, 0.47–3.26), within ≤ 24 h after induction (2.00; 95% CI, 1.11–2.88), and at a dose of < 1,000,000 (3.10; 95% CI, 1.59–4.60) (Figure 4).

**Figure 5:**
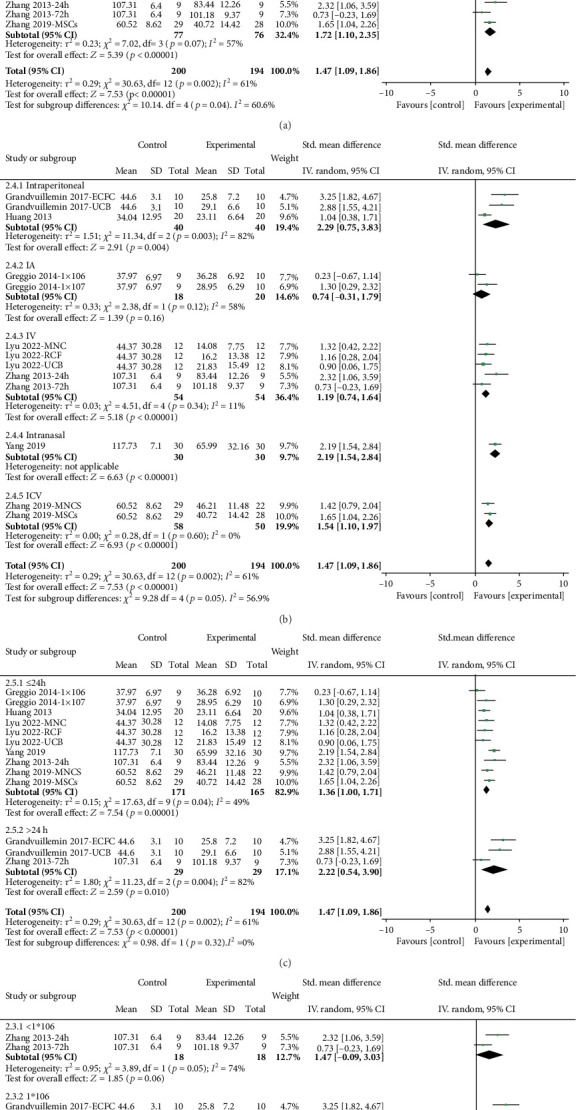
Stratification of estimated effect size for cognitive function. Forest plots demonstrating SMD and 95% CI for (a) cell type, (b) route of administration, (c) cell administration time postinjury, and (d) total cells per dose.

Cognitive: Cognitive outcomes were evaluated using the water maze test, showing a significant improvement in performance by 1.44 SMD (95% CI, 1.21–1.67; 7 studies and 13 entries; Figure 3b) with notable heterogeneity (*I*^2^ = 61.0%; *p* < 0.00001). Among the 13 interventions analyzed, 11 demonstrated SMD values exceeding 0.8, with the most substantial effect size observed at 3.25. Stratification by study design and intervention characteristics showed notable differences in effect sizes, as outlined in Figure 5. The largest effect size was observed when the cells were endothelial colony-forming cells (ECFC) (3.25; 95% CI, 1.82–4.67), administered intraperitoneally (2.29; 95% CI, 0.75–3.83), given more than 24 h after induction (2.22; 95% CI, 0.54–3.90), and at a dosage of 1,000,000 cells (1.75; 95% CI, 0.78–2.73) (Figure 5).

Overall efficacy: When pooling all studies and comparisons, there was a significant improvement in overall functional neurological outcomes improved by 1.62 SMD (95% CI, 1.08–2.14; 12 studies and 26 entries). Twenty out of the 26 interventions demonstrated SMD values exceeding 0.8, with the most substantial effect size being 5.59. High heterogeneity persisted when aggregating all entries, as evidenced by substantial variation in SMD (*I*^2^ = 90%; *p* < 0.00001).

### 3.5. Publication Bias

**Figure 6:**
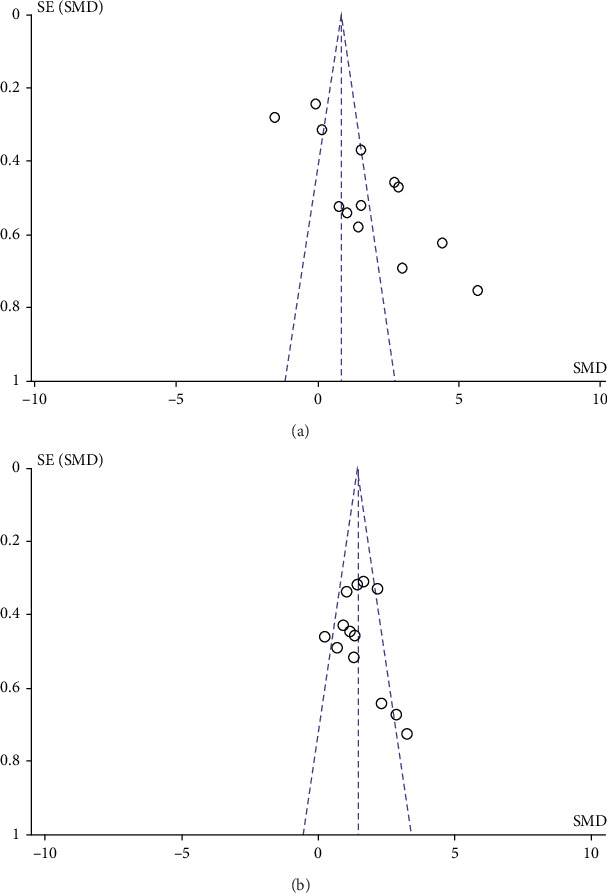
Funnel plots demonstrating publication bias from included studies. Funnel plots for (a) negative geotaxis test and (b) water maze test.

Funnel plots were constructed to assess the impact of study quality and heterogeneity on publication bias. We observed asymmetry in all funnel plots concerning sensorimotor and cognitive function, indicating the presence of publication bias in these studies (Figure 6).

## 4. Discussion

Systematic reviews are essential for translating preclinical findings into clinical applications. When coupled with meta-analyses, these reviews enable a more systematic and unbiased evaluation of experimental results. Our meta-analysis indicates that these cells exhibit promise in enhancing neurological function outcomes in neonates with HIE. Nevertheless, caution is warranted due to the overall low certainty of evidence revealed in our quality assessment.

### 4.1. The Impact of UC-Derived Cell Therapy on Neurological Function of HIE

Our examination of 12 pertinent preclinical studies through systematic review and meta-analysis revealed that UC-derived cell therapies effectively enhance neurobehavioral outcomes in sensorimotor and cognitive testing. Timothy et al. [27] conducted a similar meta-analysis of 55 preclinical studies on perinatal brain injury animal models, supporting the notion that umbilical cord blood cells therapy may improve long-term motor function through various neuromodulatory mechanisms. Our study aligns with these findings, but uniquely evaluates the efficacy of different umbilical cord cell types in HIE models. Furthermore, Archambault et al. [28] analyzed the impact of MSCs in HIE preclinical models, demonstrating a significant improvement in neurobehavioral outcomes in both sensorimotor and cognitive assessments, with an overall functional neurologic outcome improvement of 2.42 SMD. Our meta-analysis corroborates these positive effects, collectively supporting the potential utility of UC-derived cell therapy in preclinical research on neurological diseases.

This meta-analysis indicates the need for additional research to clarify the optimal dose of MSCs, as sensorimotor outcomes favored doses below 1,000,000, whereas cognitive studies favored 1,000,000. Similarly, there was inconsistency regarding the most efficacious cell type, with sensorimotor outcomes favoring Tregs and cognitive studies favoring ECFCs. These variables, among others, hold significant clinical relevance for future patient interventions. Therefore, our results should inform future investigations aimed at identifying the most favorable characteristics of UC-derived cells for achieving successful outcomes.

Our research is robust because of the thorough review of literature and strict adherence to a documented protocol, guaranteeing a careful and rigorous evaluation process. The meta-analysis incorporated information from various studies, increasing the size of the sample and accuracy when examining the effects of interest. Furthermore, the functional neurologic result's primary outcome is of great significance for future preclinical and clinical research.

### 4.2. The Identification of Knowledge Gaps

Our study revealed several knowledge gaps in the existing literature that warrant attention in future research. While HIE has been the primary focus of perinatal nervous system disease, it is important to explore the potential of UC-derived cell therapy in the treatment of other neonatal neurological conditions. Studies have demonstrated the neuroprotective effects of this approach in animal models of meningitis and intraventricular hemorrhage, suggesting its broader applicability beyond HIE. Notably, UC-derived cell therapy has demonstrated positive neuroprotective effects in mouse models of meningitis [29] and rat models of intraventricular hemorrhage IVH [30]. Our review and meta-analysis primarily concentrated on small animal models of HIE, likely due to the higher costs, time, and logistical challenges associated with large animal studies. However, future research should prioritize large animal models where feasible, as they offer greater translational value for human therapy [31]. Thus, further exploration of UC-derived cell therapy in treating other neonatal neurological diseases and in large animal models of HIE is essential.

### 4.3. Limitations

This systematic review and meta-analysis is subject to several limitations. A variety of other cell therapies sourced from diverse origins have been proposed to be effective in treating HIE. These include neural stem cells, MSCs, and endothelial progenitor cells derived from bone marrow, as well as those from other placental tissues [32, 33]. Furthermore, the research did not include trials using altered cells or those paired with supplementary therapeutic methods, such as the synthesis of other cell-based therapies or combination treatments. However, the authors acknowledge that the use of UC-derived cells in conjunction with other interventions, such as therapeutic hypothermia, may potentially offer enhanced protective effects [34]. Further, many of the included studies analyzed focused on investigating the histological or structural impacts of UC-derived cell interventions on neonatal models of HIE, with functional neurological results being considered secondarily. The diverse study designs may have influenced the outcomes of our meta-analysis. Finally, the clinical safety of umbilical cord cell therapy cannot be assessed due to the lack of comprehensive investigation into long-term effects on animal subjects in the included studies. The potential long-term repercussions are particularly worrisome in preterm neonates. Future preclinical studies should observe animal subjects that have received umbilical cord cell therapy for neonatal conditions throughout their adult life in order to determine the frequency of any negative effects. Despite these limitations, our findings are consistent with prevailing trends in this research domain. Another constraint of our study was the significant heterogeneity in treatment effects among study groups across all comparisons. Given the limited number of included studies and the possibility of selection bias, such heterogeneity is anticipated in a study of this nature. The funnel plot and effect test confirm the presence of publication bias. Furthermore, if preclinical studies do not prioritize neurological function outcomes, which serve as our primary measure, the overall study quality may be compromised. The difference in study design may be a factor in the variability observed in our analysis.

The utilization of the SYRCLE risk of bias tool reveals significant deficiencies in all the studies examined. None of the 12 studies assessed in our review was deemed to exhibit a low risk of bias based on the evaluation criteria used in the tool. It is noteworthy that only when authors explicitly disclosed these details in their published works were the reporting areas considered to have a low risk of bias. Consequently, it is plausible that these studies utilized these methods in their experiments but did not properly record them. Our analysis highlights this common deficiency and emphasizes the need for improved reporting guidelines in scholarly publications, especially in the field of preclinical translational research. We advocate for the incorporation of tools like the SYRCLE risk of bias tool during the design phase of future preclinical studies to mitigate internal reporting biases [16].

### 4.4. Future Trends

Currently, several clinical trials are ongoing involving humans, with 11 registered trials focusing on UC-derived cells in infants with HIE [35]. An example is a 2020 study that treated six full-term newborns with HIE using UC-derived cell therapy, demonstrating the safety and feasibility of the intervention [36]. With supportive findings from preclinical research, UC-derived cell therapy emerges as a promising and novel neuroprotective treatment.

## 5. Conclusion

In summary, our results indicate a promising therapeutic role for UC-derived cells in treating HIE. A meta-analysis of existing preclinical studies reveals that the administration of these cells from umbilical cord positively impacts the neurological function of treated groups, as evidenced by improvements in sensorimotor and cognitive assessments. Nonetheless, further investigation is necessary to ascertain the optimal dosage, timing, and method of administration. Despite limitations such as study reporting quality, heterogeneity, and publication bias, our findings align with current literature in this field. These factors should be considered when planning future preclinical investigations and clinical trials.

## Figures and Tables

**Table 1:** Summary of study characteristics.

**Study**	**Age injury induced**	**HUC cell type**	**Route of administration**	**Total cells per dose**	**Cell administration time postinjury**	**Duration of study**	**Comparator**
Grandvuillemin et al. [17]	PND7	UCB or ECFC	Intraperitoneal	2 × 10^7^ cells/mL (UCB), 1 × 10^6^ cells/mL (ECFC)	48 h	PND65	Injury + saline
Greggio et al. [18]	PND7	MNCs	IA	1 × 10^6^ cells/mL, 1 × 10^7^ cells/mL	24 h	PND65, 66, 67, 68, 69	Injury + vehicle
Huang et al. [19]	PND7	MNCs	Intraperitoneal	1 × 10^7^ cells/mL	24 h	PND9, PND11, PND14, PND30	Injury + saline
Lyu et al. [20]	PND7	UCB, MNC, or RCF	IV	1 × 10^7^ cells/mL	24 h	PND14	Injury + saline
McDonald et al. [21]	PND7	MNCs, EPCs, Tregs, or monocytes	Intraperitoneal	1 × 10^6^ cells/mL (MNCs), 2 × 10^5^ cells/mL (others)	24 h	PND14	Injury + PBS
McDonald et al. [22]	PND10	MSCs	Intranasal	2 × 10^5^ cells/mL	24 h	PND14	Injury + PBS
Penny et al. [10]	PND7	UCB	Intraperitoneal	1 × 10^6^ cells/mL	24 h	PND14	Injury + PBS
Penny et al. [23]	PND10	MNCs	Intraperitoneal, intranasal	1 × 10^6^ cells/mL	PND11, PND11, 13, 20	PND14	Injury + saline
Xu et al. [24]	PND10	MSCs	ICV	1 × 10^6^ cells/mL	1 h	HI 48 h or HI 28 days	Injury + PBS
Yang et al. [25]	PND7	MSCs	Intranasal	1 × 10^6^ cells/mL	30 min	PND21, PND36	Injury
Zhang et al. [26]	PND7	MSCs	IV	5 × 10^5^ cells/mL	24 or 72 h	PND36, 37, 38, 39, 40	Injury + DFB
Zhang et al. [9]	PND7	MSCs or MNCs	ICV	1 × 10^6^ cells/mL (MSCs), 1 × 10^7^ cells/mL (MNCs)	24 h	PND29, 30, 31, 32, 33, 34	Injury + PBS

## Data Availability

All datasets and analyses created in this review are available upon reasonable request.
